# A conceptual framework for interprofessional shared decision making in home care: Protocol for a feasibility study

**DOI:** 10.1186/1472-6963-11-23

**Published:** 2011-01-31

**Authors:** France Légaré, Dawn Stacey, Nathalie Brière, Sophie Desroches, Serge Dumont, Kimberley Fraser, Mary-Anne Murray, Anne Sales, Denise Aubé

**Affiliations:** 1 Centre de Recherche du Centre Hospitalier Universitaire de Québec, 10, de l'Espinay, Québec, Québec, G1L 3L5, Canada; 2 Département de Médecine Familiale et de médecine, Université Laval, Québec, Canada; 3 Faculty of Health Sciences University of Ottawa, 451 Smyth Road (Room 1480F), Ottawa, Ontario, Canada, K1H 8M5 Ottawa Hospital Research Institute, Ottawa, Ontario, Canada; 4 Centre de santé et de services sociaux de la Vieille-Capitale, 880, rue Père-Marquette, Québec, Québec, G1M 2R9, Canada; 5 Département des sciences des aliments et nutrition, Université Laval, Québec, Canada; 6 École de service social, Université Laval, Québec, G1V 0A6, Canada; 7 Faculty of Nursing, University of Alberta, 6-10L.3, University Terrace, Edmonton, Alberta, Canada; 8 Ottawa Health Research Institute, 451, Smyth Road, Ottawa, Ontario, K1N 8M5, Canada; 9 Institut national de santé publique du Québec, 945, avenue Wolfe, 5e étage Québec, Québec, Canada, G1V 5B3. Département de médecine sociale et préventive, Faculté de médecine, Université Laval, Québec, Québec, Canada

## Abstract

**Background:**

Shared decision making (SDM) is fundamental to informed consent and client-centered care. So far, SDM frameworks have been limited to the client-physician dyad, even though care is increasingly delivered by interprofessional (IP) teams. IP collaboration is especially essential in home care, one of health care's most rapidly growing areas. This study will assess whether it is possible to practice SDM in IP home care.

**Methods/Design:**

We will use a qualitative case study and a quantitative survey to capture the macro, meso and micro levels of stakeholders in home care. The case study will follow the knowledge-to-action process framework to evaluate the work of an IP home care team at a Quebec City health center. Sources of data will include one-on-one interviews with patients, family caregivers or surrogates and significant others, and administrators; a focus group of home care health professionals; organizational documents; and government policies and standards. The interview guide for the interviews and the focus group will explore current practices and clinical problems addressed in home care; factors that could influence the implementation of the proposed IP approach to SDM; the face and content validity of the approach; and interventions to facilitate the implementation and evaluation of the approach. The survey will ask 300 health professionals working in home care at the health center to complete a questionnaire based on the Theory of Planned Behaviour that measures their intentions to engage in an IP approach to SDM. We will use our analysis of the individual interviews, the focus group and the survey to elaborate a toolkit for implementing an IP approach to SDM in home care. Finally, we will conduct a pilot study in Alberta to assess the transferability of our findings.

**Discussion:**

We believe that developing tools to implement IP SDM in home care is essential to strengthening Canada's healthcare system and furthering patient-centered care. This study will contribute to the evaluation of IP SDM delivery models in home care. It will also generate practical, policy-oriented knowledge regarding the barriers and facilitators likely to influence the practice of IP SDM in home care.

## Background

Most modern healthcare systems today emphasize integrated healthcare services, patient-centered care, and the engagement of patients as partners in their own care. These three elements are key components of the strategy to improve health while keeping spending within manageable limits [1,2]. Another important element is the minimization of unwarranted variations in healthcare services, variations that are associated with high costs and problems of patient safety [3].

In this context, finding effective ways to involve patients in shared decision making (SDM) is critical. SDM is defined as a process in which healthcare professionals and patients work together to make healthcare choices: [4-7] it is considered fundamental to informed consent and patient-centered care [2,8]. SDM can improve the clinical decision-making process by reducing the overuse of options not clearly associated with benefits [9] and by enhancing the use of options clearly associated with benefits [10]. SDM is also important to patient satisfaction. A significant proportion of patients today prefer an active role in decisions concerning their health, especially when they understand expectations in this regard [11]. Patients' participation in healthcare decision making is linked to favourable patient outcomes [12] and a population-based survey has indicated that 90% to 97% of respondents do not want their physician to make decisions without their input [13]. The evidence also suggests that patients who declare their preference for low involvement in decisions are strongly influenced by self-efficacy: this suggests that given the proper knowledge and skills, patients could demand even more involvement [14]. However, only 42% to 62% of respondents to a survey of the general population said that their physician usually involved them in treatment decisions [15].

Many attempts to promote SDM in clinical practice have failed. A systematic review of surveys of healthcare professionals from 18 countries suggests that respondents perceive the three greatest barriers to implementing SDM in clinical practice as time constraints, the lack of applicability of SDM due to the patient's characteristics, and the clinical situation [16]. As interesting as these findings may be, their relevance is less obvious when we consider that of the 3231 healthcare professionals surveyed, 89% were physicians--this, even though care is increasingly planned and delivered by interprofessional (IP) teams [17-20]. The disproportionate percentage of physician respondents can be attributed to the fact that SDM models have been limited to the patient-physician dyad, even though patients make most decisions with more than one healthcare professional. It thus becomes evident that SDM models need to be reworked so as to acknowledge the involvement of multiple players. Taking this conclusion further, it becomes obvious that with Canadian healthcare policy committed to delivering patient-centered care, it is imperative that Canada foster patients' engagement in SDM with a variety of healthcare professionals, not just physicians [2,21].

There are several advantages to engaging IP teams in SDM. Interdisciplinary teams would contribute different knowledge and skills to the decision-making process, thus producing more feasible and sustainable decisions. From the patient's standpoint, an IP approach to SDM should make it even more possible for him/her to take part in decision-making, have his/her decisional needs met, and produce agreement about a healthcare treatment or option [22]. Interventions to promote an IP approach to SDM could thus improve the quality of decision support provided in the healthcare system in an integrated manner that reflects patient-centered care and improves health, while bridging the gap between professionals from various health disciplines and patients and their families, thereby reducing the silos within the healthcare system. Such interventions could also improve the decisions made by patients and their healthcare teams by fostering integrated healthcare services and the continuity of care [23] across health sectors and disciplines, and as a result of the foregoing, they could ameliorate care and better match what patients request to what they receive. Supporting these hypotheses is a Cochrane review of IP educational interventions that identified six studies, [24] of which four had positive outcomes: one on emergency department culture and patient satisfaction, another on collaborative team behaviour and the reduction of clinical error rates for emergency department teams, a third on the management of care delivered to victims of domestic violence, and the last on mental healthcare professionals' competency at delivering care. Another Cochrane review, this one of the effects of practice-based interventions to enhance IP collaboration on professional practices, concluded that practice-based IP collaborative interventions can improve healthcare processes and outcomes [25].

Our project "Interprofessional approach to shared-decision-making in primary care: Advancing theories, models, methods and measurement," funded by the Canadian Institutes of Health Research, has been underway since 2007 [22]. In the context of that study, we have contributed (i) an inventory of 15 SDM models and 24 measurement tools critically appraised for an IP approach to SDM; (ii) a theoretical analysis of the models identified [26]; and (iii) a new IP SDM model [27,28]. Our IP SDM model has been presented in details in previous publications [27,28]. Briefly, it is comprised of two main axes. The vertical axis represents the SDM process, and the horizontal axis represents individuals involved in the process. Elements at the micro level are embedded within family and IP team systems, both of which are subject to broader environmental influences.

Although our model appears to be valid for use in primary care, it has not been implemented in the context of home care. The present study will therefore assess the feasibility of using our model and its measurement tools to systematically describe the implementation of an IP approach to SDM in home care. We propose to work with a home care agency in Quebec before conducting a pilot study in Alberta to test the transferability of our study's findings to another Canadian province. We selected the area of home care because it requires IP collaboration and is growing faster than any other healthcare sector in Canada. Canada's public sector spending on home care almost doubled from 1994 to 2003, rising to $3.4 billion over nine years [29]. This rate of growth is almost twice the growth in public spending on healthcare services over this time. Based on data from the 2007 Canadian Community Health Survey (analysis conducted for this article), we found that 4.2% of Quebecers and 3.4% of Albertans received government-paid home care services in 2007. These numbers exceeded 10% among residents 65 and older in both provinces. Even more seniors received private home care, while another 4% of seniors in Quebec and 3.5% in Alberta described themselves as in need of care but unable to obtain it for a variety of reasons. In some provinces, such as Quebec, home care is coordinated through primary care, with integration and continuity of care constituting an important challenge.

A key component of home care services is the IP nature of care. In Canada, the degree to which home care is delivered by an IP team varies by jurisdiction. In both Quebec and Alberta, home care teams are made up of professionals with a variety of backgrounds: nurses, occupational therapists, physiotherapists, social workers, dieticians, and more [30]. In general, the work unit in home care agencies is a geographically based office made up of IP teams. These offices contract private agencies to provide the services deemed necessary. In planning care, consultations among healthcare professionals from different disciplines are not only common but necessary. Case managers and the IP home care team also frequently collaborate with healthcare professionals outside of the home care program, such as the patient's primary care practitioner and specialists. This collaboration is necessary to coordinate and implement the various interventions that patients require. The collaboration takes place by telephone or electronic means or in face-to-face discussions. The case manager is the pivotal professional in these exchanges.

Having explained the importance of SDM and justified our selection of home care as our area of research, our study proposes to assume the following specific objectives:

1. To identify the gap between current practices and the proposed IP approach to SDM in home care with a view to selecting decisions to target for our study and identifying the actors who help patients make decisions

2. To describe actors' perceptions of factors (i.e., barriers and facilitators) that could influence their intention to adopt an IP approach to SDM in home care

3. To develop a toolkit (e.g., a training program, educations tools, a video) to facilitate the implementation of an IP approach to SDM and overcome known barriers to implementation

## Methods/Design

### Study design

We will adopt a mixed methods design consisting of a case study and a survey. The main component will be the case study, which will be guided by the knowledge-to-action framework [31] and will follow one IP home care team at the Centre de Santé et des Services Sociaux de la Vieille-Capitale (CSSS-VC) in Quebec City. The survey will be sent to 300 IP healthcare professionals who work in home care at one of the CSSS-VC's three sites.

### Theoretical background

The knowledge-to-action framework [31] is an approach that links theory to strategic planning by first creating knowledge and then applying the knowledge through carefully chosen means. This framework will guide participants' application of new care processes, as per our original IP SDM model [27,28]. The central element of our proposed approach is the *knowledge creation *process. In previous research, we used information synthesis and consensus methods to devise an IP SDM model that proposed a new process by which healthcare teams could achieve SDM [27,28]. Now, we are prepared to translate the model into the process of care (i.e., the *action cycle*). The circular *action cycle *begins by recognizing the problem. Next, it identifies, reviews, and selects knowledge relevant to the problem. It then adapts this knowledge to the local context. Finally, barriers to knowledge use are assessed and interventions to overcome them are introduced. In subsequent phases, knowledge use is monitored, outcomes are evaluated, and sustained knowledge use is examined.

Accordingly, we will work with knowledge users (patients, family caregivers, administrators, and IP home care teams) (i) to identify decisions for which the patients and the IP teams often experience high personal uncertainty (e.g., whether the patient should move from home care to institutional care); (ii) to assess barriers and facilitators to implementing an IP approach to SDM; (iii) to develop or tailor effective implementation interventions; and (iv) to evaluate respondents' intentions to use our IP SDM model.

### Population and setting

Covering a region of 290,000 inhabitants, the CSSS-VC is a major health services organization in the Quebec City area. Between 2005 and 2008, one of the CSSS-VC's family practice sites was a pilot project for the Health Canada-funded Interprofessional Education for Collaborative Patient-Centred Practice initiative [32]. This initiative created a directorship of IP affairs within the CSSS-VC, which is currently under the leadership of a team member. However, as of the time of writing the grant application, neither the CSSS-VC nor its healthcare teams had articulated goals focusing on SDM.

Home care is one of several programs offered by the CSSS-VC. To be eligible for home care, the health issue and the patient's need must be validated by professional assessment; the patient and his/her caregivers must agree to participate in the SDM process and receive the services required; the patient must be confined to the home; it must be more efficient to offer services at home than in an outpatient setting; and the home must be safe and adequate to the patient's needs. Patients are not eligible for CSSS-VC-provided home care if they are covered by another program (e.g., car insurance or workers' compensation insurance).

#### Sampling strategies

For the case study, we will use a purposive sampling strategy to represent the macro, meso, and micro levels of care: namely, healthcare professionals, decision makers within the organization (CSSS-VC), and decision makers within regional health boards. Participants will be recruited from the following categories: (i) patients receiving home care (n = 5-8) and their caregivers (n = 5-8); (ii) administrators such as managers and policy makers who have varying influence on the home care environment (n = 4-6); and (iii) professionals from IP teams who have a significant impact on healthcare decisions (n = 5-8). Eligible healthcare professionals will include physicians, nurses, dieticians, social workers, rehabilitation therapists, respiratory therapists, pharmacists, and psychologists. Eligible patients will consist of adults with family caregivers.

For the survey, we will contact the approximately 300 healthcare professionals who provide home care for patients affiliated with one of the CSSS-VC's three sites. These patients hail from the CSSS-VC's four main groups of home care patients: patients recovering from hospitalisation, older people, end-of-life patients, and patients with mental health problems.

### Data collection tools and procedures

#### Phase 1: Identifying the factors that influence IP SDM in home care

For the case study component, we will conduct individual interviews with administrators, patients and family caregivers and will hold a focus group comprised of professionals from the IP team. In both cases, we will use a structured interview guide to appraise four elements:(i) current practice and clinical problems (i.e., decisions for which patients and IP teams feel high personal uncertainty); (ii) factors that may influence the implementation of the proposed IP approach to SDM in home care; (iii) professionals' ability to adopt our proposed IP approach to SDM when providing home care; and (iv) interventions that may help implement the approach in the home care setting.

We will present this new IP SDM approach in three ways: a live presentation, delivery of a brochure of our IP SDM model with a detailed description of its key concepts and relational statements, and a short instructional video [27,28] that depicts an IP SDM approach in home care. The main difference between the interviews with administrators and the focus group with IP team members will be the degree to which we focus on current clinical practices. The project coordinator will conduct the interviews and make field notes at the end of each interview. To ensure consistency, all interviews and focus group will be conducted by the same person. All will be audiotaped and transcribed. At the end of the interviews and the focus group, participants will be asked to answer questions about their sociodemographic characteristics.

For the survey, we will send a questionnaire based on the Theory of Planned Behaviour (TPB) to all healthcare professionals. This information will allow us to measure professionals' intentions to engage in an IP approach to SDM in home care and adapt our framework for the assessment of barriers to the implementation of SDM [16]. The TPB posits that intention is the immediate determinant of changes in behaviour [33]. It suggests that for a behaviour to change, salient beliefs underlying the factors found to be significantly associated with behavioural intention(e.g., attitude, social norms, perceived behavioural control) should be reinforced by a theory-based intervention. The TPB survey will solicit the information necessary to create interventions that increase the uptake of the desired behaviour. Our survey questionnaire will be based on validated questionnaires developed by our research team for similar projects on the implementation of SDM in clinical practice [16,34,35].

In addition to conducting a case study and a survey, we will review documents throughout the study to determine how the context at the meso and macro levels influences an IP approach to SDM at the micro level. For this, we will consult the CSSS-VC's mission statement, its description of its home care program, and other organizational documents such as newsletters, clinical protocols and regulatory documents. We will seek corroboration in exemplars discussed in the individual interviews or the focus group.

#### Phase 2: Developing a toolkit adapted to IP SDM in home care

We will refer to our analysis of the data from the interviews, the focus group, and the TPB survey to adapt our IP SDM model to the home care setting. In adapting the model, we will use evidence of effective interventions for implementing SDM in clinical practice [36] and refer to the barriers and facilitators identified in phase 1 to make a toolkit of interventions that enhance SDM and promote changes to clinical practices. The literature suggests that interventions that succeed in engaging patients to participate in health decisions include patient decision aids, educational materials, and professional training [36].

#### Phase 3: Appraising the toolkit

After developing the toolkit, we will once again work with the case study participants of phase 1 (administrators, patients, caregivers, and healthcare professionals) to (i) obtain feedback on the revised IP SDM model, (ii) reassess providers' intention to adopt an IP approach to SDM in home care, and (iii) appraise the feasibility and acceptability of the new toolkit of interventions to implement an IP approach to SDM in professionals' practice of home care. We will administer an acceptability and feasibility questionnaire using tools validated with other SDM programs [37,38]. This questionnaire will ask users to rate the amount of information supplied with each intervention, the clarity of the information in question, and the overall presentation of the intervention. It will also ask users to identify any factors that could influence implementation of the intervention.

#### Phase 4: Conducting the Alberta Pilot

Our goal in conducting an Alberta pilot is twofold: first, to ensure that the tools are translated from French to English and can thus be used in future studies conducted in English; and second, to lay the groundwork for a future study in which we will replicate the Quebec case study on a larger scale. There are two reasons why we have elected not to conduct the Quebec and Alberta study simultaneously. First, Alberta's healthcare system is undergoing substantial reorganization and we anticipate that it will be some time before we can engage decision makers and clinicians in our study. Second, it will be more effective to test our approaches in one setting and revise the tools and their implementation in light of our experience with that setting, before moving to a second venue.

To prepare for the pilot, we will translate the following tools and instruments into English: our presentation of the new IP SDM home care model for delivery by the lead facilitator, the brochure explaining the model, the video, and the TPB survey. Following translation, these instruments will be sent to the participating home care site in Alberta. The Alberta home care team is an IP team consisting of approximately 20 healthcare professionals working in a semi-rural home care office 30 kilometres south of Edmonton. One member is already working with this team to develop tools and instruments to measure case managers' workloads.

The pilot study will begin by distributing the TPB survey to the full team. Next, participants will be asked to state their understanding of the questions and their answers will be evaluated. Participants will then be assigned to a focus group and asked to listen to the presentation, read the material, and view the video. Each focus group will meet three times over 4 to 6 weeks to explore the tools. To evaluate participants' reactions to the tools, we will use the same instrument as in Quebec.

### Data analysis

#### Case Study

We expect to meet part of our primary research objective with a detailed description of our case study. Two researchers will independently analyze the content of the interview and focus group transcripts to identify evidence related to research objectives 1 and 2. This analysis will involve (i) reading each interview or focus group transcript and its field note in its entirety to obtain a sense of the overall data; (ii) conducting content analysis using the theory-based tree structure with open codes for new themes that we will derive by induction; and(iii) comparing coders' findings [39]. The unit of analysis will be the interview, with comparisons across interviews. We will pay attention to areas of consensus as well as to areas of difference. We will maintain memos of decisions and code manuals to establish an audit trail. NVivo software will facilitate our analysis.

#### Survey

In the secondary objective, we will numerically code respondents' intention to engage in an IP approach to SDM, the determinants of their intention, their acceptance of the toolkit interventions, and their sociodemographic data. All data will be entered into an Excel file. We will conduct descriptive analyses of the survey items using Excel software so as to fulfill objectives 2 and 3. We will explore differences in responses to the TPB surveys in phases 1 and 3 regarding respondents' intention to engage in an IP approach to SDM.

In a pilot clustered randomized control trial of a SDM training program, the intention of family physicians to engage in SDM was 0.8 (possible range -3 to +3) at baseline [40]. We assume that the minimal clinically significant difference in the intention of health professionals to engage in an IP approach to SDM between the phases 1 and 3 is 0.2. In order to detect an increase from 0.8 to 1.0 in the intention of health professionals to engage in an IP approach to SDM with 80% power, at a 5% significance level, one would require 126 health professionals in the study. However, it is important to consider that this present study involved health professionals from various disciplines.

#### Mixed methods: integration of qualitative and quantitative data

We will use NVivo to triangulate quantitative and qualitative findings from the different sources. To comply with rigorous methodological strategies [41], the two co-principal investigators, the co-investigators, and the research assistants associated with the home care setting will review the case study. This will generate feedback regarding the credibility of our model in the home care setting.

#### Organizational documents

Research assistants will perform content analysis of organizational documents under the leadership of a co-principal investigator. The content analysis will use a similar approach to that used to analyze the transcripts. The documents in question are expected to be a particularly important source of information, allowing us to reach our research objectives 1 and 2. We will use the notes and findings from the documents to better understand the organizational context, variations and deficiencies in current practices, and respondents' perceptions of the factors influencing an IP approach to SDM in home care.

### Ethical considerations

This research protocol was reviewed and approved by the CSSS-VC Research Ethics Board. All participants will sign consent forms approved by the CSSS-VC Research Ethics Board.

## Discussion

### Expected outcomes

To respect Canadian health policies that emphasize the need for an IP approach to patient-centered care, [42,43] it is necessary to develop tools to implement IP SDM practices in home care settings. Our IP SDM model and the support of well-trained IP healthcare teams can help home care patients become better informed, help them better understand their own health outcomes, and help them make health decisions that respect their values and preferences. This study will generate practical, policy-oriented knowledge of interventions to broaden the implementation of IP SDM in home care.

### Potential limits of the proposed research

One limitation of the proposed study is that we will only present one case. Nonetheless, our experience developing our initial IP SDM model persuades us that it is important that we advance systematically to ensure that we address barriers successfully and adapt the IP SDM model iteratively. Future work will build on the solid evidence obtained from this study and will involve multiple sites, and hence more cases, so as to ascertain the transferability of our approach across settings. The pilot project in Alberta will be an important first step in this process.

### Knowledge translation plan

This study will deliberately employ an integrated knowledge translation approach. Knowledge users will be integral members of our research team and will provide direct feedback on our new model and interventions, ensuring that these are relevant to the home care setting. The intended users of our results are healthcare teams, educators, and health policy makers interested in implementing patient-centered care processes such as an IP approach to SDM in clinical practice. We expect that many of our collaborators will use our model in the future. In addition to practicing integrated knowledge translation with our participants, at the end of our grant period, we will disseminate study results at scientific and professional conferences whose themes relate to SDM, IP, and health policy. We will also post our results on the websites of our team members' institutions of affiliation and publish our findings in open-access journals. We will disseminate key findings and the toolkit using Canadian web-based dissemination vehicles that address home care issues.

## Competing interests

The authors declare that they have no competing interests.

## Authors' contributions

FL and DS were responsible for drafting the manuscript. All authors revised and accepted the final version of the manuscript.

## Authors' information

FL is Tier 2 Canada Research Chair in Implementation of Shared Decision Making in Primary Care. FL, DS, SD, AS are members of Knowledge Translation Canada, a CIHR funded national research network. SD is a Fonds de la recherche en santé du Québec Junior 1 scholar. AS was a professor in the Faculty of Nursing at the University of Alberta and held the Canada Research Chair in Interdisciplinary Healthcare Teams (Tier 2) at the time the grant application was prepared and submitted; she is now Deputy Chief, VA Inpatient Evaluation Center, United States Department of Veterans affairs, Ann Arbor, Michigan, US. KF is a professor in the Faculty of Nursing at the University of Alberta.

## Pre-publication history

The pre-publication history for this paper can be accessed here:

http://www.biomedcentral.com/1472-6963/11/23/prepub
